# Supplementary material for: The relationship between the moon cycle and the orientation of glass eels (Anguilla anguilla) at sea
Source: R Soc Open Sci. 2019 Oct 30;6(10):190812. doi: 10.1098/rsos.190812 (PMC6837198; doi:10.1098/rsos.190812)
Supplement: Supplementary Information on the analysis, data collection, moon altitude, glass eels swimming speed and analysis of the orientation with respect to the sun. [file rsos190812supp1.docx]

The relationship between the moon cycle and the orientation of glass eels (*Anguilla anguilla*) at sea

Alessandro Cresci,^1,2*^ Caroline M. Durif,^2^ Claire B. Paris,^1^ Cameron Thompson,^2^ Steven Shema,^3^ Anne Berit Skiftesvik,^2^ & Howard I. Browman^2^

^1^Department of Ocean Sciences, Rosenstiel School of Marine & Atmospheric Science, 4600 Rickenbacker Causeway, FL 33149-1098, USA.

^2^Institute of Marine Research, Austevoll Research Station, Sauganeset 16, N-5392 Storebø, Norway.

^3^Grótti ehf., Grundarstíg 4, 101 Reykjavík, Iceland.

*Corresponding author. Email: alessandro.cresci@rsmas.miami.edu

# Supplementary Material

List of contents

**Figure S1:** Description of data collection and various steps of the analysis of circular data.

**Figure S2:** Average swimming speed of the glass eels during each moon phase.

**Figure S3.** Swimming speed of glass eels in relation to the presence of the moon above or below the horizon.

**Figure S4:** Moon zenith during the deployments at sea.

*Analysis of sun azimuth and light intensity:*

**Figure S5:** Distance between Orientation – moon azimuth by moon phase.

**Figure S6:** Distance between Orientation – sun azimuth by moon phase.

**Figure S7:** Light intensity (lum/ft^2^) in the DISC during the tests at sea.

**Figure S8:** Pairwise comparison of the light intensity data.

**Table S1:** Values of the data displayed in Fig. 4.


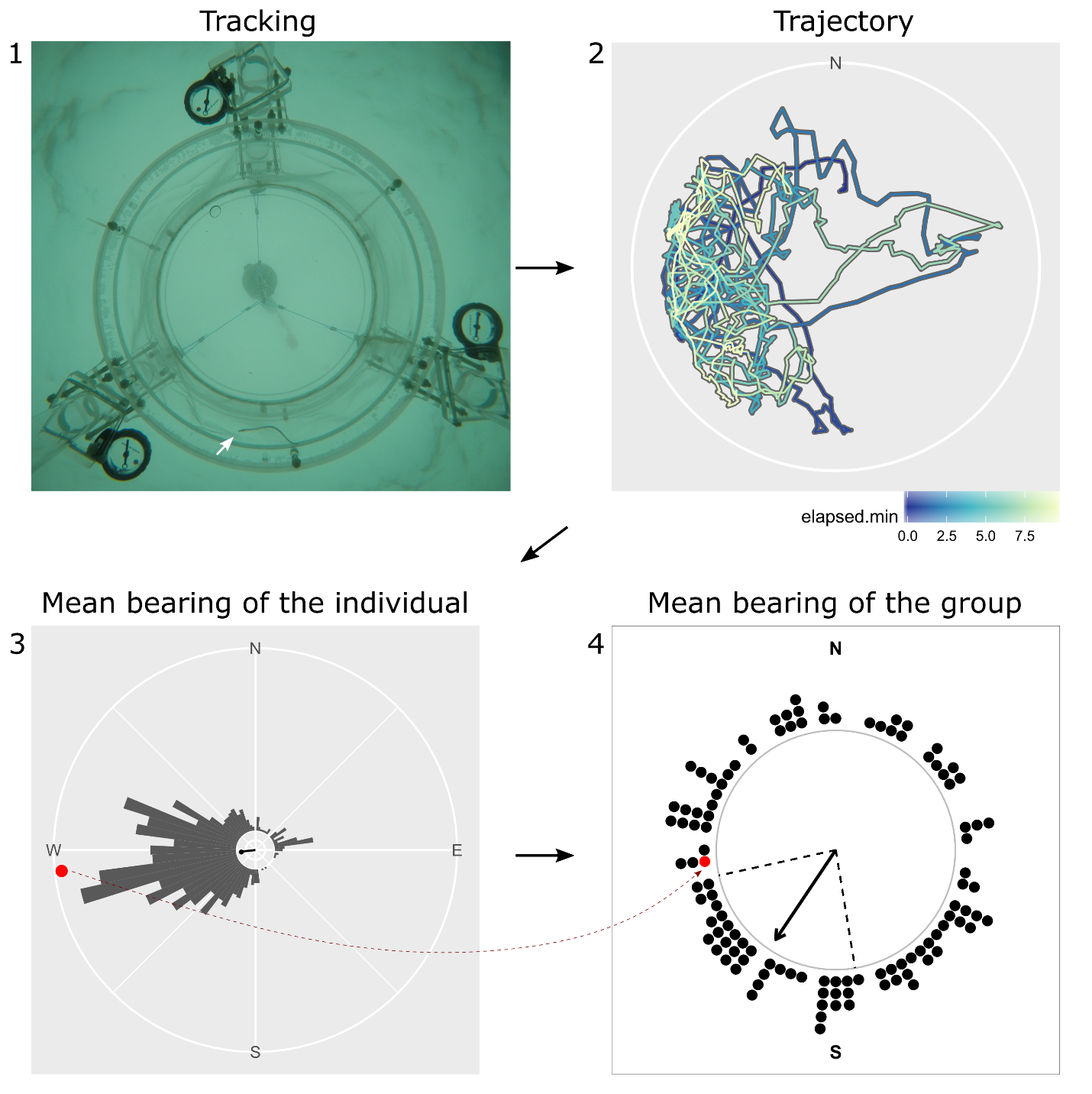


**Figure S1. Description of the analysis of the data collected with the DISC.** **1:** As first step, data on the position of the glass eel in the DISC are collected through a video tracking procedure on the videos recorded *in situ* during the deployments. The position of the head of the eel is tracked every second for 10 minutes (i.e. 600 data points are collected per each fish tested in the DISC). The photo shows an example of the view of a glass eel (highlighted by a white arrow) swimming in the behavioural chamber of the DISC. **2:** The trajectory of the larva is calculated from the datapoints collected through the video tracking. **3:** The angle of each of the 600 data points with respect to the magnetic North and the center of the chamber is considered as a bearing. Because the DISC is allowed to rotate, bearings are corrected using the digital compass that records the difference between the orientation of the camera and the magnetic North. The mean orientation of the larva is assessed applying the Rayleigh’s test of uniformity on the 600 bearings. If the outcome of the statistical test is significant (P < 0.05), the mean bearing (RED circle) is considered as the preferred orientation direction of the larva. **4:** The last step of the analysis is performed on all the preferred orientation directions of the larvae belonging to the same experimental group (i.e. at one moon phase during 1 tidal phase). The mean orientation directions of the larvae that showed a preference in the orientation is grouped and the Rayleigh’s test is applied. Through this step it’s possible to assess whether the glass eels had the tendency to orient towards a common direction (with respect of the North or with respect of the moon azimuth). In this subfigure, the RED circle corresponds to the mean orientation of the glass eel used as an example in the previous subfigure 3. In this hypothetical example glass eels had the tendency to orient SW (direction indicated by the black arrow, with the dashed lines indicating the 95% confidence intervals).


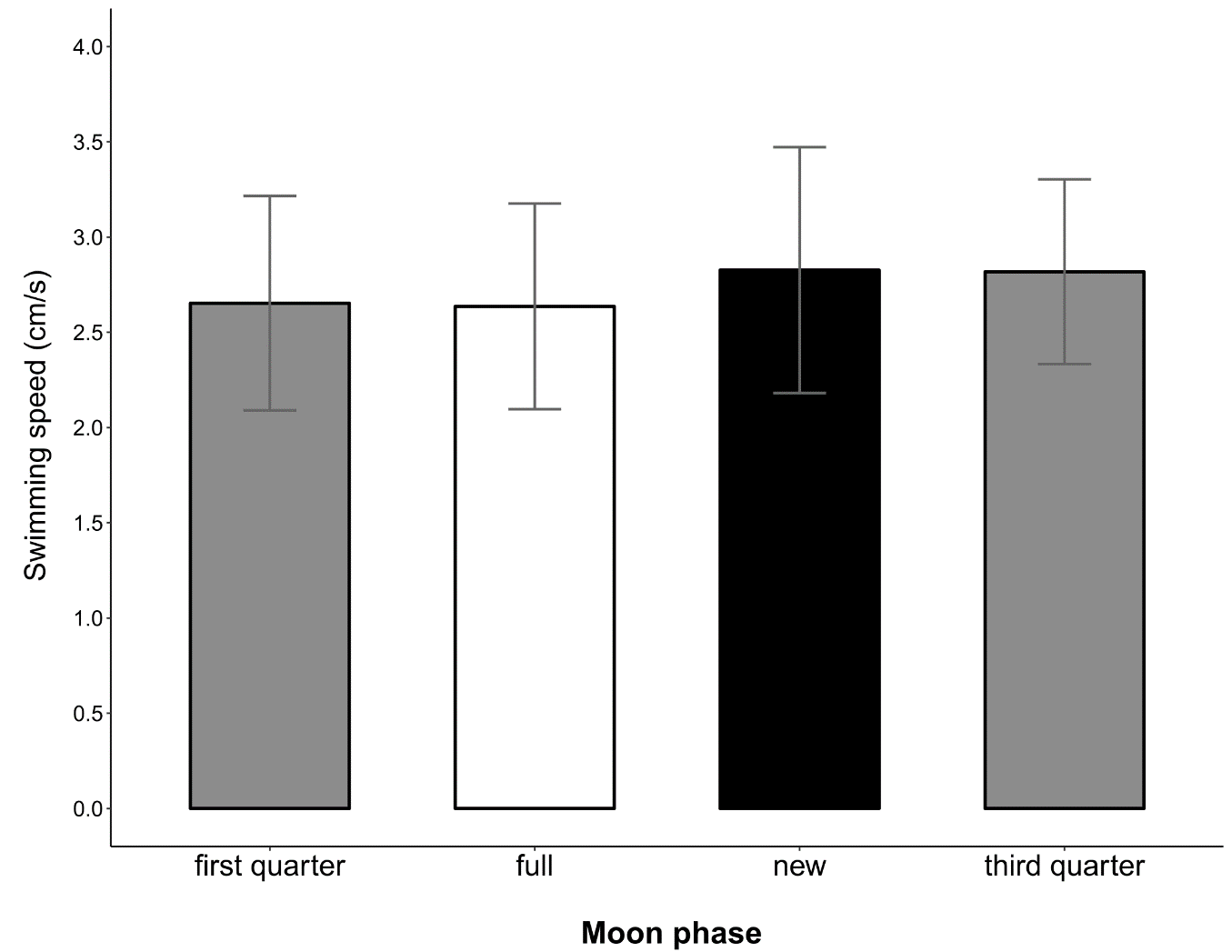


**Figure S2: Average swimming speed of the glass eels (*Anguilla anguilla*) at each moon phase.** The bars show mean ± SD swimming speed of the glass eels at each moon phase. The color of the bar displays (schematically) the level of illumination during each moon phase (white = bright moon, gray = half of the moon visibility, black = dark moon).


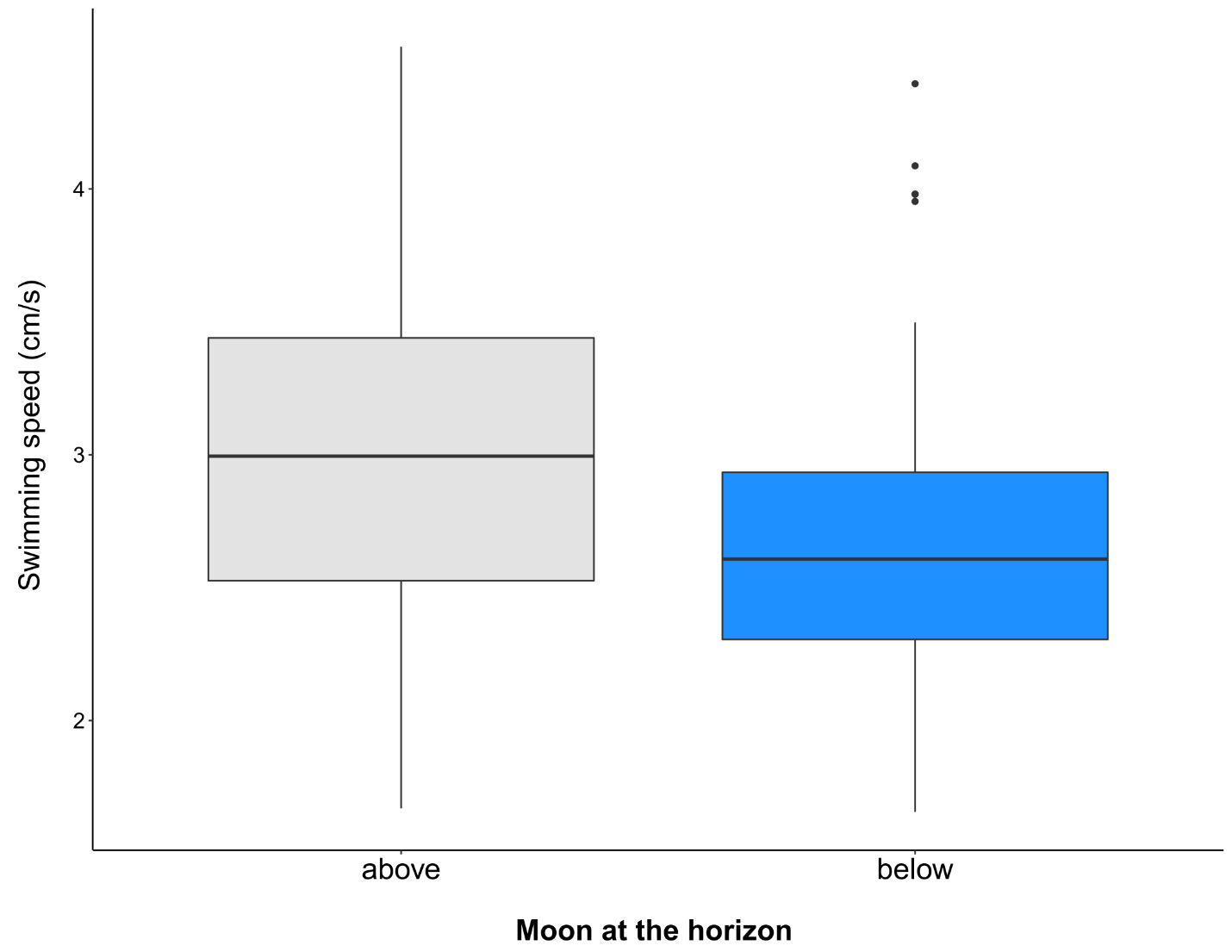


**Figure S3. Swimming speed of glass eels (*Anguilla anguilla*) in relation to the presence of the moon above or below the horizon.** The gray boxplot reports the median speed (cm/sec) of the glass eels when the moon was above the horizon, while the blue boxplot reports the median speed of the glass eels when the moon fell below the horizon.


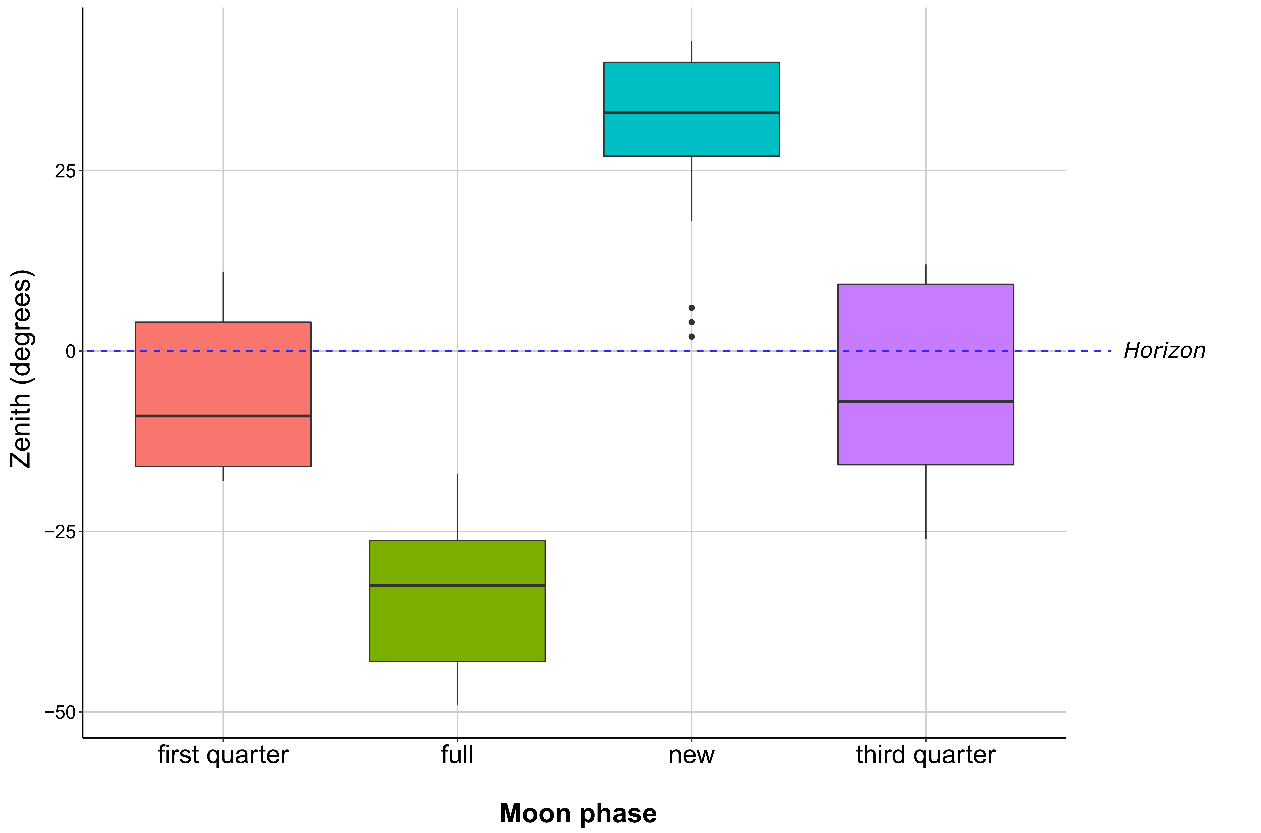


**Figure S4: Moon zenith during the deployments at sea.** The moon zenith (altitude of the moon with respect to the horizon) during the deployments at sea, at each of moon phase. The blue dashed line (y = 0°) corresponds to the line of the horizon. Negative values of the zenith angle indicate that the moon was below the horizon. Positive values of zenith indicate that the moon was above the horizon.

**2. Moon azimuth, sun azimuth and light intensity**

In the analysis described here the orientation of glass eels with respect to the azimuth of the moon and the azimuth of the sun are compared.

In Figure S5 and Figure S6, data on the distance between the orientation directions of the glass eels and the azimuth of the moon and the sun (top of each circular plot) are displayed. In both analyses data are grouped according to the moon phase. Figure S5 shows the same data reported in Figure 4.iii (main text) but pooled according to the moon phase only.

Significant patterns in the orientation with the moon are observed only during new moon and less precisely during first quarter (Fig. S5) (as we also describe in the manuscript). Interestingly, the exact same patterns are observed with respect to the sun: the eels appear to orient towards the sun at full moon and first quarter (Fig. S6).

At new moon, the moon is above the horizon and its average azimuth direction is South. However, the average direction of the sun azimuth is always South as well, during any lunar phase. Thus, following the moon at new moon will also result in a significant orientation with respect to the sun. However, the two celestial bodies have the same average direction during this moon phase.

If the eels were using the sun as an orientation cue, we would expect to observe sun-related orientation independent of the moon phase, as the sun does not change properties with the lunar phase. However, the sun-related orientation disappears at full moon and third quarter, when the sun is above the horizon, but the moon is not (Fig. S4), and the light intensity does not differ significantly from that present during new moon. We compared the average illumination levels (HOBOs) in the DISC (Fig. S7) between the moon phases to see whether the differences in orientation behaviour could be related to differences in light intensity levels during the tests (the light comes from the illumination of the sun during daytime). There was no difference in illumination between full, third quarter and new moon (Fig. S8), although there was an important difference in orientation behaviour of the eels between these 3 phases. This supports the hypothesis that the orientation of the glass eels does not depend on solar illumination, and indicates that the sun-related orientation at new moon is an artifact caused by the eels orienting towards the moon during this lunar phase.


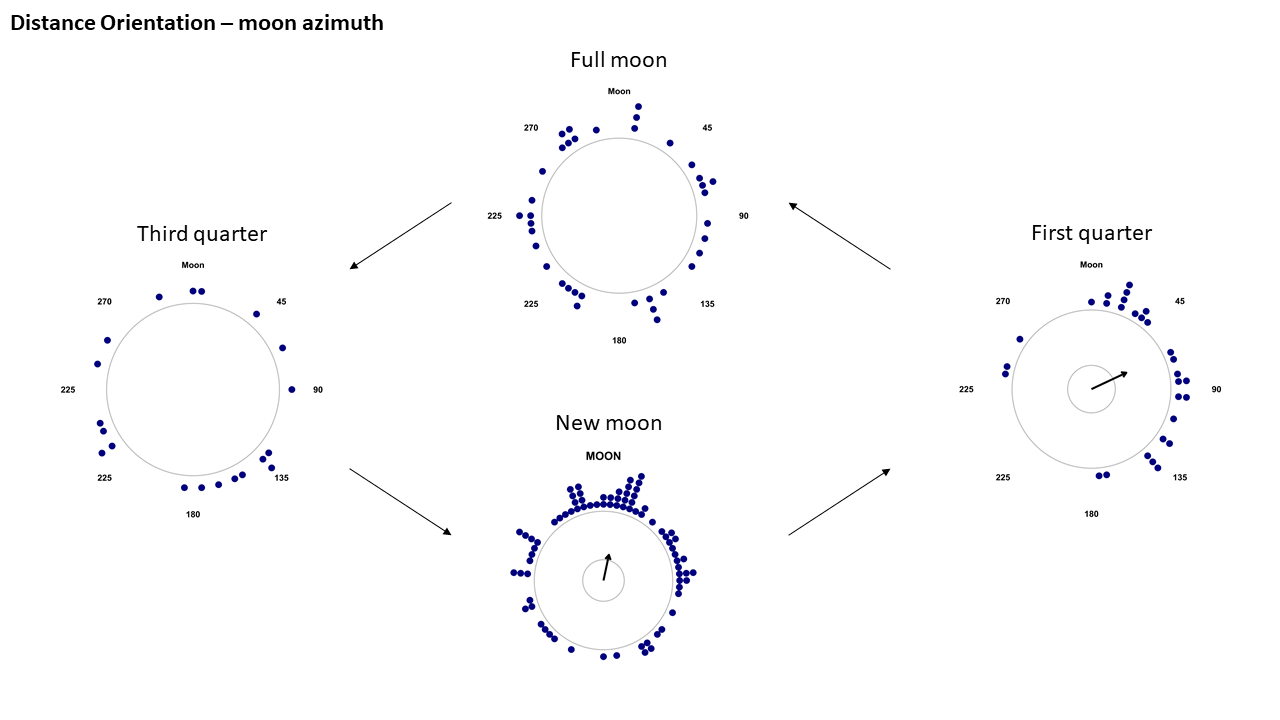


**Figure S5: Distance between Orientation – moon azimuth by moon phase.** The angular distance between the orientation direction of each glass eel (*Anguilla anguilla*) (1 data point = 1 glass eel) and the direction of the moon azimuth (top of each circle at the time that each animal was observed at sea in the DISC). Arrows in the center of the circle shows significant (Rayleigh’s p < 0.05) patterns in the orientation direction of the eels. The direction of the arrow shows the mean orientation direction and the length of the arrow the accuracy of that orientation (Rayleigh’s r). Empty circles show no patterns in the orientation (Rayleigh’s p > 0.05).

**
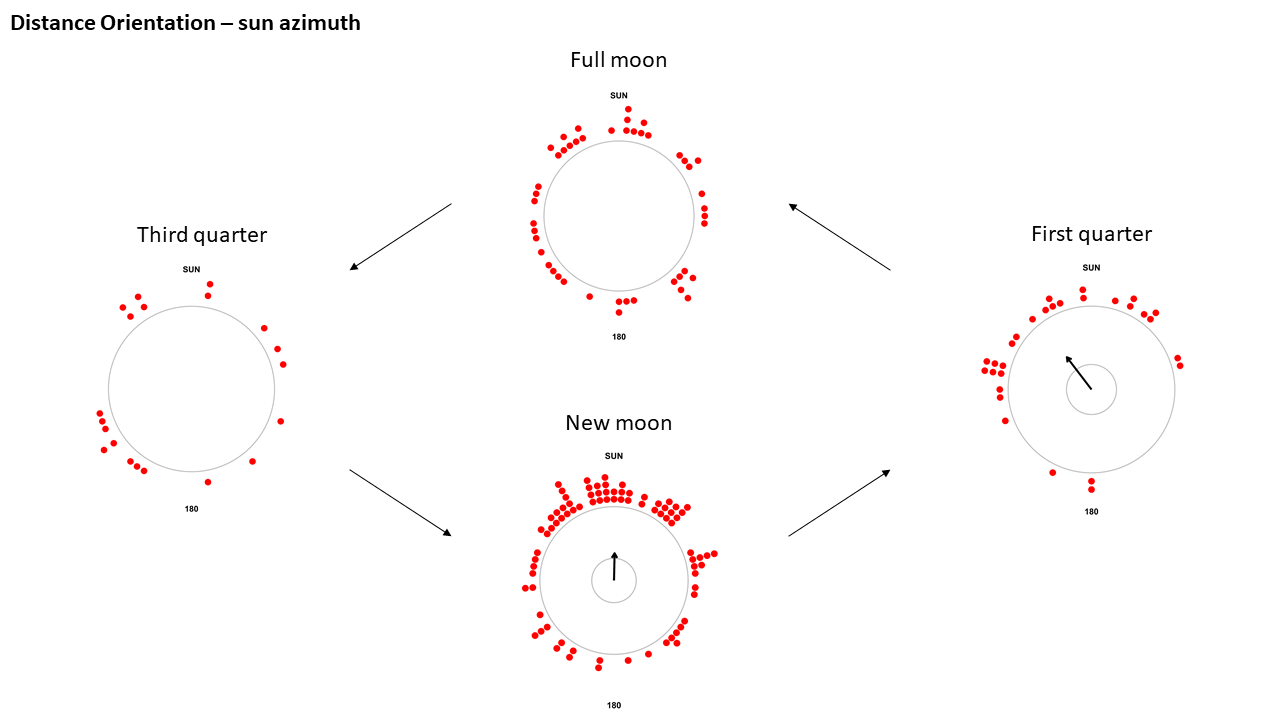
**

**Figure S6: Distance between Orientation – sun azimuth by moon phase.** The angular distance between the orientation direction of each glass eel (*Anguilla anguilla*) (1 data point = 1 glass eel) and the direction of the sun azimuth (top of each circle) at the time that each animal was observed at sea in the DISC. The features of the plots are the same as described in Figure S3.


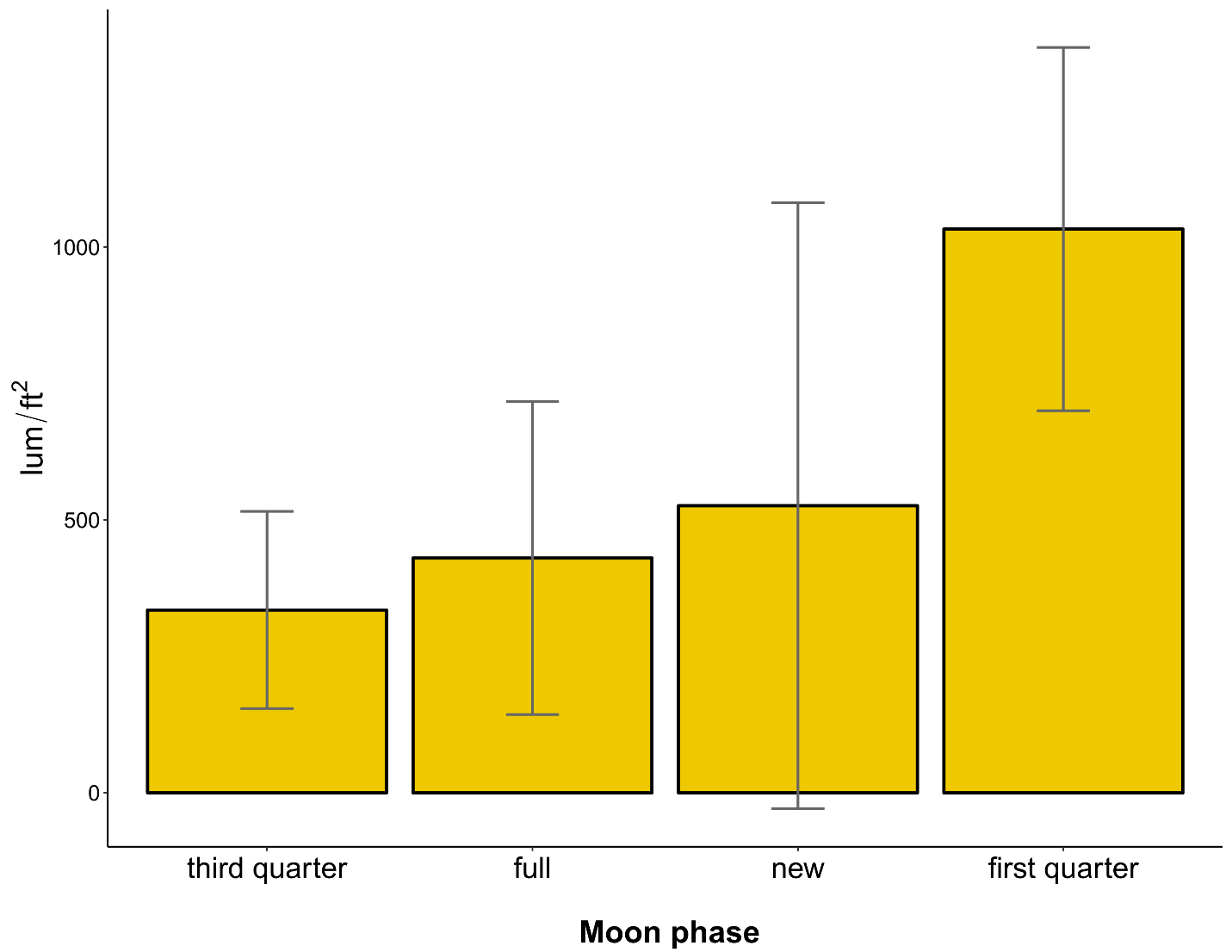


**Figure S7: Light intensity (lum/ft^2^) in the DISC during the tests at sea (mean ± SD).**


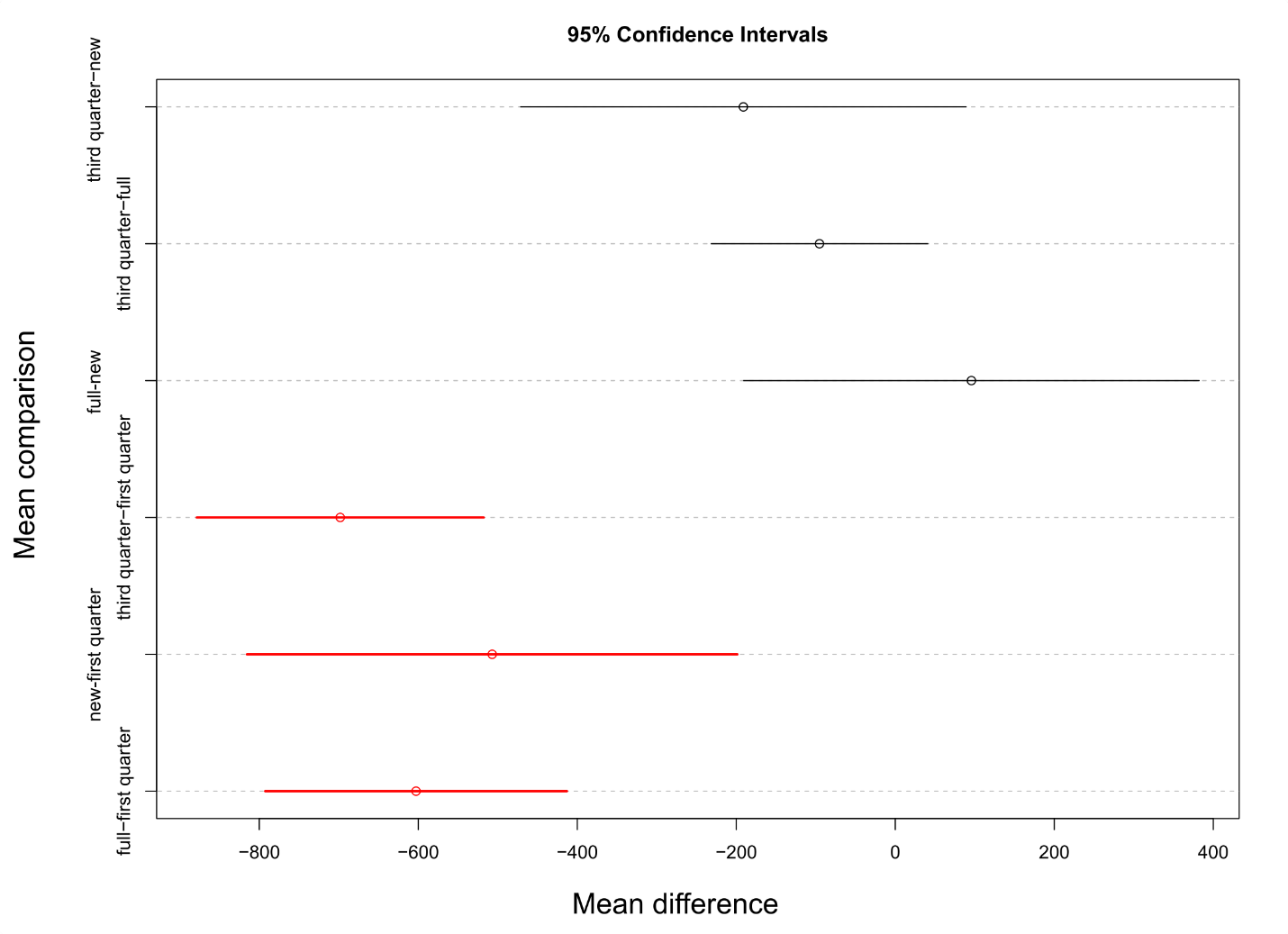


**Figure S8: Pairwise comparison of the light intensity data shown in Figure S5.** The Tukey-Kramer Pairwise Multiple Comparison for the light intensity data (alpha = 0.05). Horizontal lines with circles are the mean (± SD) differences of light intensity between two moon phases (listed on the y axis). Red lines show significant differences in light intensity; grey lines show no significant difference in light intensity.

**Table S1:** Values of the data displayed in Fig. 4. Tide, moon phase, position of the moon at the horizon and the angles ɑ_north_ and ɑ_moon_ are listed for each eel that displayed orientation.

| **Tide** | **Moon phase** | **horizon** | **ɑ_north_** | **ɑ_moon_** |
| --- | --- | --- | --- | --- |
| ebb | full | below | 163.6 | 150.6 |
| ebb | full | below | 86.7 | 68.7 |
| ebb | full | below | 306.2 | 282.2 |
| ebb | full | below | 301.1 | 271.1 |
| ebb | full | below | 0.0 | 324.0 |
| ebb | full | below | 157.8 | 116.8 |
| ebb | full | below | 10.0 | 328.0 |
| ebb | full | below | 53.7 | 7.7 |
| ebb | full | below | 17.0 | 328.0 |
| flood | full | below | 293.7 | 7.7 |
| flood | full | below | 349.6 | 62.6 |
| flood | full | below | 251.7 | 321.7 |
| flood | full | below | 345.2 | 53.2 |
| flood | full | below | 261.3 | 325.3 |
| flood | full | below | 143.0 | 206.0 |
| flood | full | below | 99.7 | 158.7 |
| flood | full | below | 36.7 | 94.7 |
| flood | full | below | 217.2 | 271.2 |
| flood | full | below | 340.5 | 34.5 |
| flood | full | below | 21.0 | 70.0 |
| flood | full | below | 322.9 | 7.9 |
| flood | full | below | 206.1 | 249.1 |
| flood | full | below | 121.5 | 207.5 |
| flood | full | below | 128.4 | 210.4 |
| flood | full | below | 41.5 | 123.5 |
| flood | full | below | 269.7 | 343.7 |
| flood | full | below | 88.1 | 161.1 |
| flood | full | below | 150.9 | 220.9 |
| flood | full | below | 92.4 | 160.4 |
| ebb | full | below | 227.6 | 232.6 |
| ebb | full | below | 103.2 | 106.2 |
| ebb | full | below | 218.3 | 217.3 |
| ebb | full | below | 275.2 | 266.2 |
| ebb | full | below | 183.0 | 170.0 |
| ebb | full | below | 91.0 | 76.0 |
| ebb | full | below | 317.9 | 297.9 |
| ebb | full | below | 282.6 | 260.6 |
| ebb | full | below | 134.7 | 162.7 |
| ebb | full | below | 156.2 | 178.2 |
| ebb | full | below | 252.0 | 273.0 |
| ebb | full | below | 213.1 | 228.1 |
| ebb | full | below | 232.8 | 245.8 |
| ebb | full | below | 219.6 | 228.6 |
| ebb | full | below | 214.7 | 220.7 |
| ebb | full | below | 293.3 | 295.3 |
| ebb | full | below | 71.1 | 71.1 |
| flood | third quarter | below | 131.7 | 246.7 |
| flood | third quarter | below | 169.8 | 283.8 |
| flood | third quarter | below | 344.5 | 91.5 |
| flood | third quarter | below | 49.7 | 156.7 |
| flood | third quarter | below | 69.1 | 173.1 |
| flood | third quarter | below | 260.5 | 0.5 |
| flood | third quarter | below | 331.2 | 66.2 |
| flood | third quarter | below | 205.5 | 298.5 |
| flood | third quarter | below | 97.7 | 186.7 |
| flood | third quarter | below | 317.4 | 40.4 |
| flood | third quarter | below | 283.0 | 4.0 |
| ebb | third quarter | above | 332.6 | 136.6 |
| ebb | third quarter | above | 346.2 | 150.2 |
| ebb | third quarter | above | 74.4 | 233.4 |
| ebb | third quarter | above | 183.6 | 339.6 |
| ebb | third quarter | above | 81.0 | 236.0 |
| ebb | third quarter | above | 12.9 | 162.9 |
| ebb | third quarter | above | 347.6 | 132.6 |
| ebb | third quarter | above | 345.1 | 129.1 |
| ebb | third quarter | above | 106.1 | 248.1 |
| flood | new | above | 95.8 | 357.8 |
| flood | new | above | 329.7 | 229.7 |
| flood | new | above | 305.4 | 203.4 |
| flood | new | above | 119.5 | 15.5 |
| flood | new | above | 355.4 | 248.4 |
| flood | new | above | 329.4 | 221.4 |
| flood | new | above | 195.0 | 84.0 |
| flood | new | above | 33.1 | 277.1 |
| flood | new | above | 106.2 | 345.2 |
| ebb | new | above | 163.6 | 16.6 |
| ebb | new | above | 129.1 | 341.1 |
| ebb | new | above | 244.7 | 92.7 |
| ebb | new | above | 210.8 | 67.8 |
| ebb | new | above | 257.0 | 100.0 |
| ebb | new | above | 166.0 | 7.0 |
| ebb | new | above | 307.8 | 143.8 |
| ebb | new | above | 321.9 | 150.9 |
| ebb | new | above | 100.2 | 286.2 |
| ebb | new | above | 184.2 | 6.2 |
| ebb | new | above | 167.5 | 345.5 |
| ebb | new | above | 202.0 | 18.0 |
| ebb | new | above | 252.4 | 63.4 |
| ebb | new | above | 182.4 | 350.4 |
| ebb | new | above | 90.4 | 255.4 |
| ebb | new | above | 260.1 | 62.1 |
| ebb | new | above | 240.1 | 39.1 |
| ebb | new | above | 234.4 | 31.4 |
| flood | first quarter | above | 100.0 | 37.0 |
| flood | first quarter | above | 105.4 | 42.4 |
| flood | first quarter | above | 189.6 | 122.6 |
| flood | first quarter | above | 137.0 | 70.0 |
| flood | first quarter | above | 211.8 | 141.8 |
| flood | first quarter | above | 195.6 | 124.6 |
| flood | first quarter | above | 160.2 | 87.2 |
| flood | first quarter | above | 104.1 | 30.1 |
| flood | first quarter | above | 142.3 | 65.3 |
| flood | first quarter | above | 254.5 | 176.5 |
| flood | first quarter | above | 117.3 | 36.3 |
| flood | first quarter | above | 167.9 | 85.9 |
| flood | first quarter | below | 53.1 | 358.1 |
| ebb | first quarter | below | 125.7 | 111.7 |
| ebb | first quarter | below | 318.2 | 304.2 |
| ebb | first quarter | below | 39.7 | 21.7 |
| ebb | first quarter | below | 37.6 | 18.6 |
| ebb | first quarter | below | 118.2 | 94.2 |
| ebb | first quarter | below | 302.5 | 278.5 |
| ebb | first quarter | below | 199.5 | 171.5 |
| ebb | first quarter | below | 169.0 | 140.0 |
| ebb | first quarter | below | 53.6 | 21.6 |
| ebb | first quarter | below | 40.5 | 7.5 |
| ebb | first quarter | below | 55.6 | 17.6 |
| ebb | first quarter | below | 50.0 | 8.0 |
| ebb | first quarter | below | 126.1 | 80.1 |
| ebb | first quarter | below | 143.0 | 97.0 |
| ebb | first quarter | below | 337.6 | 286.6 |
| ebb | first quarter | below | 191.9 | 140.9 |
| ebb | new | above | 276.2 | 134.2 |
| ebb | new | above | 115.0 | 328.0 |
| ebb | new | above | 131.3 | 338.3 |
| ebb | new | above | 94.8 | 297.8 |
| ebb | new | above | 175.0 | 12.0 |
| ebb | new | above | 246.5 | 77.5 |
| ebb | new | above | 148.6 | 332.6 |
| ebb | new | above | 189.9 | 7.9 |
| ebb | new | above | 128.2 | 300.2 |
| ebb | new | above | 213.6 | 20.6 |
| ebb | new | above | 214.3 | 17.3 |
| ebb | new | above | 278.9 | 75.9 |
| flood | new | above | 47.7 | 298.7 |
| flood | new | above | 258.1 | 145.1 |
| flood | new | above | 49.7 | 290.7 |
| flood | new | above | 134.8 | 12.8 |
| flood | new | above | 129.5 | 1.5 |
| flood | new | above | 162.8 | 29.8 |
| flood | new | above | 222.2 | 84.2 |
| ebb | new | above | 159.2 | 17.2 |
| ebb | new | above | 236.0 | 88.0 |
| ebb | new | above | 176.7 | 342.7 |
| ebb | new | above | 251.5 | 51.5 |
| ebb | new | above | 295.5 | 91.5 |
| ebb | new | above | 296.2 | 86.2 |
| ebb | new | above | 37.1 | 182.1 |
| ebb | new | above | 179.6 | 318.6 |
| ebb | new | above | 158.7 | 292.7 |
| ebb | new | above | 216.0 | 346.0 |
| flood | new | above | 274.9 | 18.9 |
| flood | new | above | 315.8 | 56.8 |
| flood | new | above | 54.2 | 151.2 |
| flood | new | above | 35.7 | 128.7 |
| flood | new | above | 64.1 | 326.1 |
| flood | new | above | 95.5 | 353.5 |
| flood | new | above | 85.2 | 339.2 |
| flood | new | above | 165.0 | 55.0 |
| flood | new | above | 350.5 | 236.5 |
| flood | new | above | 138.4 | 19.4 |
| flood | new | above | 62.7 | 299.7 |
| flood | new | above | 147.2 | 18.2 |
| flood | new | above | 195.2 | 62.2 |
| ebb | new | above | 51.7 | 273.7 |
| ebb | new | above | 122.0 | 339.0 |
| ebb | new | above | 228.2 | 11.2 |
| ebb | new | above | 136.6 | 273.6 |
| ebb | new | above | 302.2 | 75.2 |
| ebb | new | above | 43.4 | 171.4 |
| ebb | new | above | 103.4 | 226.4 |
| flood | new | above | 359.6 | 114.6 |
| flood | new | above | 274.8 | 17.8 |
| flood | new | above | 152.5 | 251.5 |
| flood | new | above | 290.7 | 26.7 |
